# Spoken Language Change in Children on the Autism Spectrum Receiving Community-Based Interventions

**DOI:** 10.1007/s10803-022-05511-4

**Published:** 2022-03-24

**Authors:** David Trembath, Matt Stainer, Teena Caithness, Cheryl Dissanayake, Valsamma Eapen, Kathryn Fordyce, Veronica Frewer, Grace Frost, Kristelle Hudry, Teresa Iacono, Nicole Mahler, Anne Masi, Jessica Paynter, Katherine Pye, Shannon Quan, Leanne Shellshear, Rebecca Sutherland, Stephanie Sievers, Abirami Thirumanickam, Marleen F. Westerveld, Madonna Tucker

**Affiliations:** 1 Griffith University, Southport, QLD Australia; 2 La Trobe University, Bundoora, VIC Australia; 3 University of New South Wales, Sydney, NSW Australia; 4 St Giles Society, Launceston, TAS Australia; 5 Murdoch Children’s Research Institute, Melbourne, VIC Australia; 6 Anglicare, Prospect, SA Australia; 7 University of Canberra, Bruce, ACT Australia; 8 University of Adelaide, Adelaide, SA Australia; 9 Griffith University, Griffith Institute for Educational Research, Mount Gravatt, QLD Australia; 10 AEIOU, Woolloongabba, QLD Australia

**Keywords:** Autism, Minimally verbal, Intervention, Communication, Language, Preschool

## Abstract

We assessed the spoken language of 73 preschool aged children on the autism spectrum receiving community-based early intervention at two time points, approximately 7 months apart. Using the Spoken Language Benchmarks, there was a small non-significant change in the proportion of children transitioning from below, to at or above, Phase 3 (word combinations). Using binomial regression, a model comprising seven of nine clinician-proposed child-related predictors explained 64% of the variance. None of the predictors were individually significant, although a large effect size (OR = 16.71) was observed for children’s baseline rate of communicative acts. The findings point to substantial unmet clinical need in children with minimal verbal language, but also the relevance of clinician-proposed predictors of their spoken language outcomes.

## Introduction

There is evidence of substantial heterogeneity in the course of early spoken language development in children on the autism spectrum, with a large minority entering school with little or no functional speech, often despite intervention (Brignell et al., 2018; Norrelgen et al., 2015). These children are variously described in the research literature as being prelinguistic, non-verbal, or minimally verbal (Koegel et al., 2020), or as having complex communication needs (Light et al., 2019). Irrespective of the term used, the impacts of children having spoken language difficulties can be far reaching, and include negative effects on learning and participation during childhood and into adult life (Cummins et al., 2020). Accordingly, it is essential that factors that may influence children’s spoken language outcomes are identified, with the view to tailoring interventions to children’s individual strengths and needs. It is important, however, to highlight at the outset that spoken language is just one mode of communication used alongside other means (e.g., gesture) and, like all people, children on the autism spectrum should be supported and encouraged to use a range of modes of their choosing, including augmentative and alternative communication (AAC) systems. Nevertheless, spoken language is highly effective in helping people communicate in spontaneous and flexible ways for a variety of purposes, including expressing thoughts, emotions, wants, and needs in everyday interactions and settings, and thus was the focus of this study.

A range of factors have been identified as possible predictors of spoken language outcomes in later childhood for children on the autism spectrum including broad developmental characteristics (e.g., chronological age, autism characteristics, non-verbal cognition, and receptive language), more specific social-cognitive (e.g., joint attention, play, imitation) and linguistic capacities (e.g., phonetic inventory), and social-contextual factors (e.g., parental social-economic status) (Chenausky et al., 2018; Mouga et al., 2020; Thurm et al., 2007, 2015; Wodka et al., 2013). However, as noted by Pecukonis et al. (2019) in a review of 21 studies examining social-communication factors, inconsistencies in participant characterisation, research design, predictor and outcome construct selection and measurement, and analytic approach make it difficult to draw conclusions that can directly inform clinical practice. One way to enhance the relevance of findings is to evaluate factors that clinicians themselves believe to be relevant to children’s spoken language outcomes and embedding these in research within community settings.

While inherently challenging for controlled design, embedding research in community settings offers the opportunity to build an empirical evidence base with direct relevance to clinical practice and rapid translational potential. Toward this end, (Trembath et al., 2021) undertook a qualitative study to delineate clinician-proposed predictors of spoken language outcomes for children on the autism spectrum. The sample of 14 speech pathologists, each working in early intervention settings, together identified 183 factors, including a range of child autism-specific and broader developmental characteristics (e.g., cognitive ability), specific social-cognitive factors (e.g., prelinguistic skills), and the presence of co-occurring conditions. Drawing on these clinical insights and the published research evidence more broadly, we designed the current quantitative study to examine the potential predictive value of selected factors for spoken language outcomes of children on the autism spectrum who had minimal spoken language at the point of intake into community-based early intervention programs.

The current study was a clinical-research collaboration between clinical staff working in seven community-based early intervention centres and researchers across six universities, developed in response to the clinician-identified need to better understand and support communication development in children enrolling at the centres with minimal spoken language. All aspects of the study methodology, including selection of factors that would be examined, were designed in accordance with an evidence-based practice framework, which combines the best available research evidence with evidence derived from clinical practice, along with the preferences and priorities of fully informed clients (Sackett et al., 1996). Accordingly, nine factors were selected through discussion involving the clinical representatives of each early intervention centre and the researchers in the team, that involved consideration of the following criteria: (a) identified relevance by speech pathologists engaged in community practice (Trembath et al., 2021), (b) clear theoretical relevance to spoken language development, (c) existing empirical evidence for a potential association with spoken language outcomes in children on the autism spectrum, and (d) capacity for feasible measurement during semi-structured play-based assessment undertaken in the context of community-based early intervention settings.

Three broad developmental factors—child *chronological age, autism characteristics*, and *receptive language*—were included given their identified clinical relevance at entry to early intervention programs and frequent inclusion in published empirical research on spoken language outcomes for children on the autism spectrum. While the latter has yielded inconsistent evidence of predictive value (possibly due to differences in study sample and design characteristics; Pecukonis et al., 2019), strong theoretical rationales for each of these as potential determinants of spoken language outcome (e.g., Mundy & Neal, 2000; Towle et al., 2020) further justifies their inclusion in ongoing research.

Two social-cognitive and social-communicative factors were selected. *Functional use of objects* reflects an underlying organization of thought and behaviour enabling children to learn from interaction with objects in their environment (Lyytinen et al., 1997, 1999). Empirically, this has been shown to predict change in children’s communication skills, including in longitudinal naturalistic observational studies (e.g., Poon et al., 2012), and to moderate intervention outcomes (e.g., Yoder & Stone, 2006). Children’s *rate of communicative acts* was selected based on theory and evidence that the propensity to direct communicative vocalizations, verbalizations, or gestures towards others is a strong predictor of both later verbal and nonverbal outcomes (e.g., Plumb & Wetherby, 2013; Shumway & Wetherby, 2009). Clinically, this propensity for children to communicate with others and the way they engage with objects (e.g., toys, equipment, learning materials) is directly relevant to their learning and participation in early childhood education settings.

Finally, four factors specifically concerning child language development were selected. Children’s *range of communicative functions* was selected based on longstanding evidence that children on the autism spectrum show a relative reduction in the pragmatic use of language for socially-motivated versus more instrumental functions (Wetherby, 1986; Wetherby & Prutting, 1984), thereby reducing the range of opportunities for spoken language to be used. *Symbolic word learning*—the ability to infer associations between spoken object labels, pictures of objects, and actual objects in the environment without explicit teaching—was selected given it is fundamental to communication development (Allen & Lewis, 2015), combined with emerging empirical evidence to suggest it may be impaired in some children on the autism spectrum (Allen & Lewis, 2015; Hartley & Allen, 2015; Rose et al., 2020). *Phonetic inventory*—the number of different speech sounds produced—is a core aspect of structural language development in children but impaired in at least some children on the autism spectrum (Saul & Norbury, 2020; Yoder et al., 2015). The *ratio of speech to non-speech vocalizations* was selected on the conceptual basis that it reflects children’s capacity to produce intelligible—and thus easily interpreted—communicative acts, and emerging evidence of a possible association with greater language development among children receiving intervention (e.g., Plumb & Wetherby, 2013; Trembath et al., 2019).

## Study Aims

The study aims were to document changes in spoken language in a cohort of preschool aged-children on the autism spectrum receiving community-based early intervention and examine the potential relevance of nine clinician-proposed predictors in accounting for children’s outcomes. Using the Spoken Language Benchmarks (Tager-Flusberg et al., 2009) to measure children’s language, we addressed two research questions: (a) what proportion of children who entered intervention at Phase 1 or 2 (*preverbal communication* or *first words*) progressed to Phase 3 (*word combinations*) at follow-up approximately 7-months later, and (b) to what extent were children’s outcomes explained by baseline differences in their developmental/cognitive capacities and social-contextual characteristics. Nine months was the anticipated average time between assessments conducted at intake (from January onwards) to the November–December of the same calendar year. We hypothesised that greater *functional use of objects, rate of communicative acts, range of communicative functions, phonetic inventory*, and *ratio of speech to non-speech vocalizations*, along with the presence of *symbolic word learning* capacity, would be associated with children attaining Phase 3 (word combinations) or higher. The potential predictive value of *chronological age*, *receptive language*, and *autism characteristics* were also explored, but with no specific hypotheses delineated due to the mixed findings in previous research.

## Methods

### Study Design and Setting

A prospective longitudinal cohort design was used to examine changes in the spoken language of children on the autism spectrum who were newly enrolled at one of seven community-based early intervention centres across six states of Australia. “The centres provided specialised early intervention support for preschool children (ages 0–6 years) with autism spectrum disorder (ASD) and ASD like characteristics, as identified by community healthcare professionals. Parents enrolled their children directly, without the need for a referral. Intervention approaches and models varied across the centres, including primarily behavioral interventions, naturalistic developmental behavioral interventions (i.e., Early Start Denver Model, Social Communication, Emotional Regulation and Transactional Support, Treatment and Education of Autistic and Related Communication Handicapped Children, and a combination of intervention practices consistent with an eclectic approach to selection (Odom et al., 2012).

### Recruitment and Participant sample

At each site, a research manager asked a speech pathologist on staff to identify children who, upon intake to the centre, would likely meet the criteria for Phase 3 (word combinations) or below on the Spoken Language Benchmarks. This phase equates to having a maximum expressive language level of approximately 30 words, early 2-word combinations, simple phonotactic structures with a limited inventory of approximately 10 consonants, and small range of communicative functions (Tager-Flusberg et al., 2009). No additional inclusion/exclusion criteria were applied to ensure a clinically relevant sample. The research manager then invited parents of these children to participate and obtained informed consent. Seventy-three children aged 20–67 months at intake (16 female, 57 male) were recruited to the study, and their characteristics are summarized in Table 1.

**Table 1:** Participant characteristics at Time 1 assessment

	Centre^a^						Total
	A	B	C	D	E	F	
No. children	10	12	18	10	10	13	73
*Chronological age*^b^							
Mean	47.50	31.08	48.06	46.10	41.90	33.77	41.53
SD	(7.65)	(7.29)	(10.34)	(9.22)	(4.79)	(7.47)	(10.54)
Min–Max	[36–57]	[20–41]	[32–67]	[35–64]	[32–47]	[21–44]	[20–67]
*Gender*							
Male	10	10	13	7	8	9	57
Female	0	2	5	3	2	4	16
*SCQ total score*							
Mean	22.30	17.58	17.11	15.83	15.63	19.36	18.23
SD	(5.93)	(4.89)	(6.05)	(7.55)	(7.05)	(6.23)	(6.29)
Min–Max	[11–30]	[11–24]	[9–26]	[5–24]	[7–25]	[6–30]	[5–30]
*Vineland adaptive behaviour Scales—2*^c^							
*Communication (standard score)*							
Mean	53.80	68.36	61.50	56.50	59.29	66.60	61.57
SD	(12.35)	(9.86)	(9.87)	(12.28)	(13.73)	(18.72)	(13.61)
Min–Max	[36–74]	[58–85]	[42–78]	[40–74]	[44–87]	[44–87]	[23–92]
*Daily living skills S (standard score)*							
Mean	56.78	73.82	68.20	56.33	65.14	74.20	66.81
SD	(10.79)	(13.43)	(11.35)	(12.26)	(13.99)	(10.55)	(13.52)
Min–Max	[48–81]	[61–99]	[53–89]	[43–73]	[53–89]	[58–85]	[43–99]
*Socialization (standard score)*							
Mean	57.78	71.36	69.30	62.00	65.43	71.70	66.89
SD	(9.82)	(7.34)	(11.57)	(12.18)	(13.60)	(11.90)	(11.65)
Min–Max	[44–79]	[63–82]	[57–92]	[46–75]	[53–94]	[52–86]	[44–94]
*Motor skills (standard score)*							
Mean	68.89	81.45	73.80	69.17	69.29	93.80	77.21
SD	(5.73)	(10.74)	(10.32)	(9.00)	(12.75)	(13.93)	(13.88)
Min–Max	[61–78]	[71–108]	[61–88]	[59–81]	[56–91]	[68–111]	[56–111]
*Adaptive behavior composite (standard score)*							
Mean	56.11	70.45	65.00	58.33	62.14	74.20	65.23
SD	(7.15)	(9.23)	(7.89)	(10.21)	(12.48)	(12.25)	(11.47)
Min–Max	[47–71]	[63–91]	[54–77]	[46–72]	[50–88]	[46–89]	[46–91]
*CSBS*							
*Social composite (standard score)*							
Mean	5.30	3.92	8.67	4.70	4.20	8.77	6.29
SD	(3.09)	(1.31)	(4.83)	(2.16)	(2.57)	(4.40)	(4.02)
Min–Max	[3–12]	[3–6]	[3–16]	[3–9]	[3–11]	[3–16]	[3–16]
*Speech composite (standard score)*							
Mean	9.70	7.50	8.39	7.90	8.90	8.69	8.48
SD	(3.20)	(2.43)	(3.52)	(2.96)	(3.54)	(4.17)	(3.33)
Min–Max	[6–15]	[3–12]	[3–14]	[3–13]	[4–17]	[3–15]	[3–17]
*Symbolic Composite (standard score)*							
Median	4	3	5	3	3.5	9	4
IQR	(1.75)	(1.00)	(6.75)	(0.75)	(1)	(3)	(5)
Min–Max	[3–10]	[3–8]	[3–12]	[3–10]	[3–10]	[3–14]	[3–14]
*Total (standard score)*							
Median	69	65	85	67.5	66.5	94	71
IQR	(27)	(4.5)	(8.5)	(17.5)	(5.25)	(29)	(27)
Min–Max	[65–110]	[65–93]	[55–122]	[12–86]	[65–115]	[65–119]	[12–122]
*MacArthur-Bates CDI*							
*Words understood*							
Median	22	106	102	125	62.5	159	102
IQR	(128.5)	(92)	(204.5)	(248.25)	(40.25)	(154.25)	(170)
Min–Max	[0–202]	[11–295]	[10–390]	[24–375]	[22–245]	[8–317]	[0–390]
*Words produced*							
Median	6.5	9	67	34	10	35.5	15
IQR	(37.5)	(24.5)	(128.5)	(151.25	(20.75)	(143.25	(75)
Min–Max	[0–202]	[0–51]	[0–356]	) [0–317]	[0–182]	) [0–295]	[0–356]

^a^The purpose of this study was not to compare outcomes for children attending the different centres. Accordingly, the centres are de-identified and the children attending the two sites for Centre C sites have been combined

^b^An ANOVA with post-hocs showed that children were younger in Centres B and F than all other centres. No other differences in age were observed

^c^All centres used caregiver report form, except F which used the structured caregiver interview form

### Assessment Procedure and Measures

A comprehensive assessment battery was completed with each child to establish phase attainment against the Spoken Language Benchmarks (Tager-Flusberg et al., 2009) and appraise capacities on the nine putative predictors. The battery included two measures of children’s communication skills (Caregiver completed: MacArthur-Bates—Communicative Development Inventories—2nd Ed, MB-CDI, Fenson et al., 2007; Clinician delivered: Communication and Symbolic Behavior Scales—Developmental Profile, CSBS-DP, Wetherby et al., 2002), a semi-structured language sampling protocol (Clinician delivered: Eliciting Language Samples for Analysis—Toddler version, ELSA-T; Barokova et al., 2020), and experimental object play and symbolic word learning tasks. Data were also collated for two assessments completed by service centre staff as part of routine intake assessments: the caregiver-completed Social Communication Questionnaire (SCQ; Rutter et al., 2003) and Vineland Adaptive Behaviour Scales (VABS-2; Sparrow et al., 2005). Language samples from the ELSA-T were transcribed using the Systematic Analysis of Language Transcripts, NZAU version (SALT; Miller et al., 2017).

Child intake assessments were completed at enrolment to the centre (February to June 2018) and repeated at exit (November/December). Herein we refer to these as Time 1 and Time 2 respectively. Assessment tasks were administered by a research assistant (external clinician or where internal, a staff member not directly responsible for the children they assessed) with clinical/allied health training and experience working with young children on the autism spectrum. The tasks were typically completed in one session, unless centre programming required a shortened session or the assessor made a clinical judgement that administration should be split over two sessions (e.g., based on children’s fatigue or declining engagement). Each session began with the administration of the ELSA-T—a 15–30-min semi-structured play-based protocol designed to elicit natural language samples—followed by the CSBS and object play and symbolic word learning tasks (with flexible order of these to maximize child engagement). Clinicians attempted all assessments where possible, but abandoned administration if children showed dissatisfaction or distress consistent with ethical mandates and routine clinical practice.

### Outcome Measure

The primary study outcome was change in children’s communication phase according to Tager-Flusberg et al. (2009)’s Spoken Language Benchmarks. Phase 1 refers to the *Pre-Verbal Communication* stage and generally applies to typically developing children aged 6–12 months showing pre-verbal intentional communication (i.e., babbling, gesture use) but no spoken language. At Phase 2 (*First Words*; typically 12–18 months), children use single words referentially and symbolically with some intelligible speech including early consonant sounds. At Phase 3 (*Word Combinations*; typically 18–30 months), children join 2 or 3 words together with an increasing vocabulary size. At Phase 4 (*Sentences*; typically 30–48 months), children combine words into sentences including some morphological markers and at Phase 5 (*Complex Language*, typically beyond 48 months), children have extensive vocabularies and communicate in grammatically complex sentences. Each child’s phase level at Time 1 and Time 2 was determined by drawing on data from the ELSA-T transcription, MacArthur-Bates CDI, and Mullen Scales of Early Learning assessment scores. To be credited with having attained a particular phase, the child had to satisfy the criteria for each domain (i.e., phonology, vocabulary, grammar, and pragmatics) based on at least one measure, as specified by Tager-Flusberg et al. (2009).

### Predictor Measures

#### Broad Child Characteristics

Child chronological age was determined from the day of CSBS-DP completion. Receptive language was also taken from the CSBS-DP, operationalised as the child’s standard score on the ‘understanding’ domain. Characteristics of autism were operationalised as the total score on the caregiver-completed Social Communication Questionnaire.

#### Functional Use of Objects

Children’s functional use of objects was calculated based on a 5-min video recorded sample of spontaneous engagement with a set of 13 common objects. For each object the child picked up, use was coded as either destructive (e.g., throwing, breaking), touching/holding, conventional/appropriate (e.g., turning pages of book, placing party hat on head), or pretend/imaginative (citation withheld for blind review). The proportion of functional use was calculated by dividing the sum of ‘touch/hold, conventional/appropriate, and pretend/imaginative’ use by the total number of observed behaviours, multiplied by 100. Reliability was evaluated through double coding of a randomly selected 22 (30.14%) of object play task clips. Behavioral observations for which there was agreement were divided by the total number of agreements and disagreements, multiplied by 100 to yield mean raw agreement across samples of 89.57% (range 0.25–1.00). Note that agreement of 0.25 for one comparison was an outlier, with the next lowest score being 0.63.

#### Rate of Communicative Acts

Each child’s rate of communicative acts was measured from item 4 of the CSBS-DP, which quantifies the number of communicative acts produced across six communication opportunities. Acts are defined as gestures, vocalizations, or verbalizations that are directed towards the adult as signals serving a communicative function. For each opportunity, the assessing clinician recorded a maximum of three acts with the mean number of acts produced by each child retained for analysis.

#### Symbolic Word Learning

The extent to which children demonstrated evidence of symbolic (i.e., referential) word learning was assessed using procedures outlined by Allen and Lewis (2015) in which children are shown photos of unfamiliar objects (i.e., d-clamp) that the examiner labels using a non-word (e.g., “dax”). The presentation and labelling of target objects is alternated with the presentation of photos of distractor objects, which are also verbally labelled. During a mapping test, the examiner then presents the child with a photo of the target object and the real object and asks the child to “give me the dax”, and identification of the target object (as opposed to the photo of the object) is taken as demonstration of referential (symbolic) learning. Scoring reliability was evaluated by double coding of a randomly selected 21 (28.77%) video recordings of task administration following the same procedure as described above for functional use of objects. Mean raw agreement here was 97.56% (range 0.00 to 1.00). The score of 0 was for one participant and related to the disagreement between the examiner and rater regarding task administration. This participant did not have complete data for all predictor measures, and so was not included in the regression analyses.

#### Range of Communicative Functions

The number of different communicative functions expressed via spoken language was calculated for each child based on communicative acts observed during the ELSA-T language sample. Research assistants (speech pathologists) reviewed video recordings and coded the function of each potentially communicative act produced by each child following the definitions provided by Wetherby (1986): request for object, action, social routine, permission, information; response to question, direction; protest; acknowledgement; showing off; comment; self-regulation; label; performative; exclamatory. Reliability via consensus coding indicated 230 instances of disagreement for coding of communicative functions across a total sample of 7,518 transcribed and coded utterances, yielding 96.94% raw agreement.

#### Phonetic Inventory

The number of different consonants and vowels produced by each child was assessed using the Phonetic Inventory for Singleton Consonants and Vowels from the Children’s Independent and Relational Phonological Analysis: Australian English 2 (Baker, 2017). This was determined by research assistants (speech pathologists) based on video recordings of children’s speech production during the ELSA-T semi-structured language sample. Reliability checks were completed via double-coding of a randomly selected 17 samples (23.3%), using Cohen’s Kappa to assess phoneme by phoneme agreement. Mean agreement across samples was 0.569 (range 0.343 to 0.739), representing fair to moderate agreement (Altman, 1999).

#### Ratio of Speech to Non-Speech Vocalizations

The percentage of speech-like vocalizations compared to non-speech vocalizations produced by each child was also derived from recordings of the ELSA-T language sampling using Behavioral Observation Research Interactive Software (BORIS; Friard & Gamba, 2016). Specifically, the amount of time (seconds) during which each child engaged in the production of speech-like vocalizations was totalled and divided by the amount of time spent engaging in both speech-like and non-speech like behaviour. Reliability for speech/non-speech vocalizations was calculated for a randomly selected 5 (7.2%) recordings using the in-built Cohen’s Kappa calculator within BORIS. The calculator compared if the two raters agreed on the presence/absence of speech and non-speech behaviours at 1-s intervals. Mean agreement across samples was 0.471 (range 0.359 to 0.596), indicating fair to moderate reliability (Altman, 1999).

### Analyses

Data were prepared in Microsoft Excel and then transferred to R Studio Version 1.2.5019 (running R version 3.6.1) for analysis. Descriptive statistics were used to determine the proportion of children at intake and outcome who met criteria for Phase 3 or above on the Spoken Language Benchmarks (Research Question 1). Data were missing for 6 children who exited the early intervention programs prior to Time 2, leaving a sample of 67. A McNemar’s test was used to test for consistency in children’s phase level across the two time points (< 3 versus ≥ 3) on the Spoken Language Benchmarks. We used odds-ratio to quantify effect size, with cut-offs of 1.68, 3.47, and 6.71 as indicative of small, medium and large effect sizes, respectively (based on Chen et al., 2010).

Binomial logistic regression was used to examine the extent to which the nine baseline characteristics predicted differences in spoken language changes (Research Question 2). Analyses were restricted to 43 children for whom complete data were available using pairwise deletion and who had (a) not attained Phase 3 Spoken Language Benchmarks at intake; and (b) were aged at least 30 months at outcome assessment (i.e., the age at which children typically achieve Phase 3;Tager-Flusberg et al., 2009). Spearman’s correlations revealed a high degree of multicollinearity (variables included receptive language, rate of communicative acts, symbolic word learning, phonetic inventory, percentage speech-like vocalisations; see Table 2), leading to the exclusion of two variables on theoretical grounds, whereby their relevance to speech production was considered in relative rather than absolute terms, in comparison to other putative predictors. Functional use of objects was removed on the basis that it reflects underlying organisation of thought and behaviour while the percentage of speech-like vocalizations on the basis that it represents the most distal of the set of behaviors involved in verbal production of language, from comprehension through to speech production. The model met the assumptions for linearity of predictors (i.e., between predictors and logit values) and multicollinearity (all VIF factors < 3.02), and there were no influential outliers (indicated by standardised residuals and Cook’s distance). The model residuals were normally distributed. We used *R*^2^ as our measure of effect size for the proportion of variability that could be attributed to the model.

**Table 2:** Means, standard deviations, and correlations with confidence intervals of predictor variables

Variable	*M*	*SD*	1	2	3	4	5	6	7	8
1. Chronological age	41.45	10.49								
2. Receptive language	5.55	3.30	− .04							
			[− .30, .23]							
3. ASD characteristics	14.38	9.51	− .14	− .03						
			[− .39, .12]	[− .29, .24]						
4. Functional use of objects	0.89	0.19	− .02	.23	− .01					
			[− .28, .24]	[− .03, .47]	[− .27, .26]					
5. Rate of communicative acts	1.87	0.89	− .02	.54**	.04	.02				
			[− .28, .25]	[.33, .71]	[− .23, .30]	[− .25, .28]				
6. Symbolic word learning	0.18	0.39	− .04	.48**	− .11	.25	.32*			
			[− .30, .23]	[.24, .66]	[− .36, .16]	[− .02, .48]	[.07, .54]			
7. Number of communicative functions	5.61	3.57	− .01	.09	.16	− .05	.11	− .05		
			[− .27, .25]	[− .18, .34]	[− .10, .41]	[− .31, .22]	[− .16, .36]	[− .31, .21]		
8. Percent of speechlike vocalisations	61.26	34.53	.14	.10	− .18	− .15	− .05	− .25	.12	
			[− .14, .39]	[− .17, .36]	[− .43, .09]	[− .40, .12]	[− .32, .22]	[− .48, .02]	[− .15, .38]	
9. Phonetic Inventory	22.27	9.41	− .00	.01	.26	− .11	.16	− .17	.37**	.28*
			[− .26, .26]	[− .26, .27]	[− .00, .49]	[− .37, .15]	[− .11, .40]	[− .41, .10]	[.12, .58]	[.02, .51]

*M* and *SD* are used to represent mean and standard deviation, respectively. Values in square brackets indicate the 95% confidence interval for each correlation. The confidence interval is a plausible range of population correlations that could have caused the sample correlation (Cumming, 2014)

*Indicates *p* < .05.

**Indicates *p* < .01. The mean value of 0.18 for Symbolic Word Learning reflects a pass rate of 18% for this task

## Results

### Changes in the Proportion of Children with Minimal Verbal Language

**Fig. 1:**
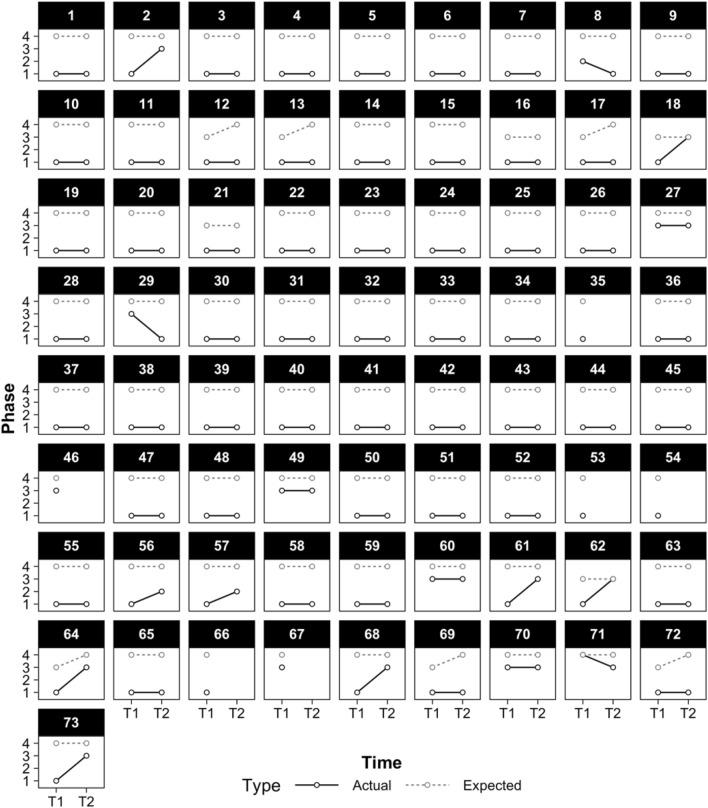
Expected and actual phase levels for the 73 children using the spoken language benchmarks at two time points

The first study aim was to identify the proportion of children who entered the early intervention programs with minimal verbal language and then went on to develop phrase level speech at the outcome assessment. Assessments were on average approximately 7 months apart (less than anticipated 9-month follow-up period) due to some children enrolling in programs later in the year (M = 222.4 days, SD = 27.55, range 144–300 days). Figure 1 presents the expected phase level (based on chronological age) and assessed phase level for all 73 children who enrolled in the study. Only one of these children (1.4%) was at age level expectations at Time 1, consistent with recruitment focusing on children with minimal spoken language, and only 2 out of 67 (3.0%; 6 children were not recorded at T2) were at age level expectations at Time 2. In descriptive terms, of the 67 children for whom data were available at both time points, 55 children (82%) maintained their phase level while 9 children (13%) showed improvement, and 3 children (4%) were assessed to have regressed with respect to phase level.

**Fig. 2:**
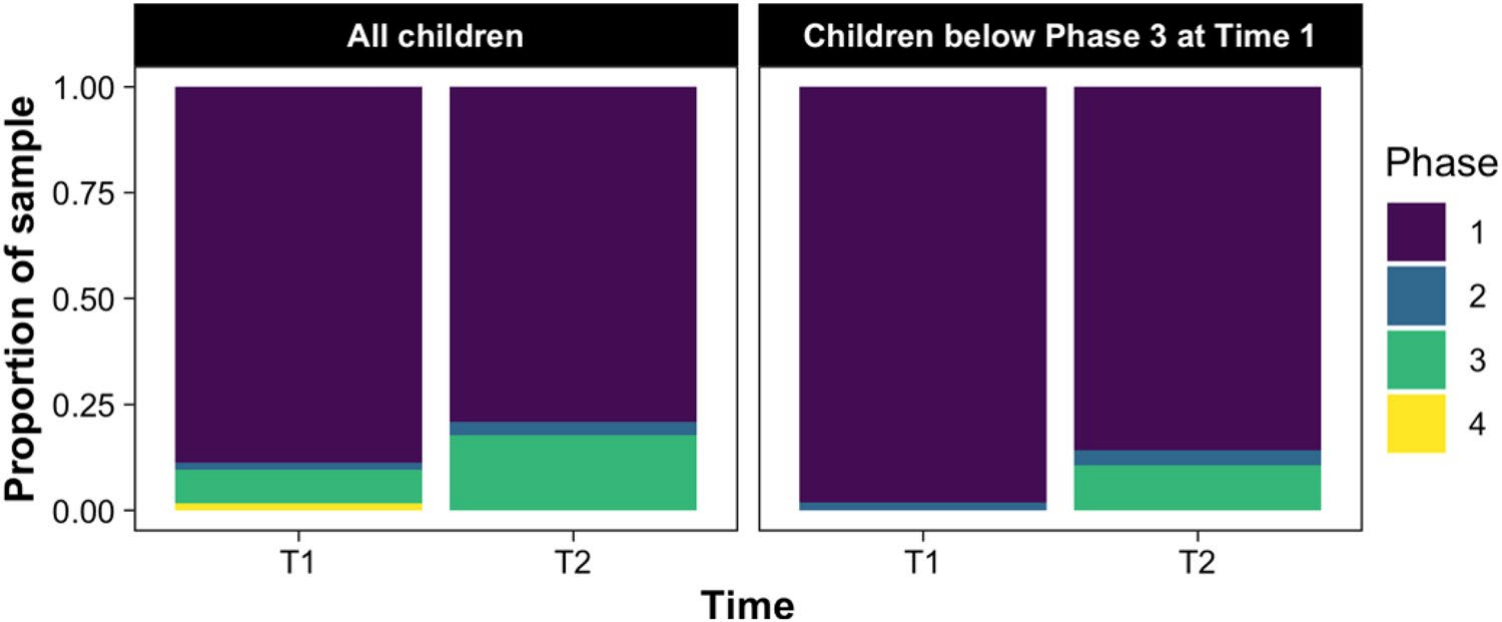
Changes in the proportion of children meeting criteria for Phase 3 of the spoken language benchmarks from Time 1 to time 2

As illustrated in Fig. 2, there was an increase in the proportion of children who met criteria for Phase 3 of the Spoken Language Benchmarks, from six children (9%) at Time 1 to 12 children (18%) at Time 2. A total of 7 of the 61 children (11%) who were below Phase 3 at Time 1, met criteria for Phase 3 at Time 2, whereas one child no longer met Phase 3 criteria, representing a small, non-significant effect for the group overall χ^2^(1) = 3.125, *p* = 0.078, odds-ratio = 1.95). When limited to only children (*n* = 62) who were at least 30 months of age at Time 2 (i.e., beyond the chronological age window for Phase 3), six children (10%) were found to be at Phase 3 of the Spoken Language Benchmarks Time 1, increasing to 11 children (18%) at Time 2, representing a small, non-significant effect (χ^2^(1) = 2.286, *p* = 0.131, odds-ratio = 1.79). Thus overall, as a group, children did not significantly change in their language phase over time, although some individual children showed improvements.

### Predictors of Change in Spoken Language

The second study aim was to examine the extent to which nine factors predicted change, where observed, in children’s spoken language. Using binomial logistic regression with the seven retained putative predictors, the model was statistically significant χ^2^ (7) = 19.94, *p* = 0.006*,* and explained 64% of the variance. Examination of *p*-values across 1000 bootstrapped samples given the reduced, and hence small, sample size yielded an average significant effect (*p* = 0.026, 95% CI = 0.019–0.032). As presented in Table 3, none of the individual predictors was statistically significant, although the rate of communicative acts had a large effect size (OR = 16.71, Z = 1.71, *p* = 0.087).

**Table 3:** Logistic regression examining predictors of acquisition of phrase level speech in children who entered early intervention with minimal verbal language

Predictor	Estimate	SE	*Z*	*p*	*sr*^2^
Intercept	− 3.65	7.74	− 0.47	0.637	
Chronological age	− 0.14	0.14	− 1.00	0.315	0.18
Receptive language	0.40	0.73	0.55	0.585	0.04
ASD characteristics	− 0.30	0.32	− 0.94	0.347	0.17
Rate of communicative acts	2.82	1.65	1.71	0.087	0.41
Symbolic word learning	− 23.93	4957.15	− 0.00	0.996	0.25
Phonetic inventory	0.18	0.21	0.86	0.387	0.08
Number of communicative functions	− 0.17	0.39	− 0.42	0.671	0.05

Estimates represent the log odds of having achieved Phase 3 at Time 2 = 1 vs having achieved phase 3 at Time 2 = 0. Specificity of model = 0.96, Sensitivity = 0.80. Odds ratios can be interpreted as effects of small (1.68), medium (3.47) and larger (6.71) based on Chen et al. (2010)

## Discussion

Our first aim in this study was to identify the proportion of children who entered the community-based early learning centres with minimal spoken language, who progressed to develop spoken language. The results are somewhat sobering, in that we observed only a small increase in the number of children (7 of 61 children) below Phase 3 at Time 1 who then went on to meet criteria for Phase 3 at Time 2. Only two children moved from below age-expected phase level to age-expected phase level over the course of the study, while three children were assessed to have regressed from Time 1 to Time 2. These findings are consistent with previous research (e.g., Norrelgen et al., 2015; Rose et al., 2020; Wodka et al., 2013) in demonstrating variability in outcomes, including promising change for some children, but substantial ongoing communication needs for many more children on the autism spectrum. From a clinical perspective, the variability in children’s outcomes over the study period should serve to support parents and clinicians in calling for individualised approaches to promoting the communication development of children on the autism spectrum who commence early intervention with minimal verbal language. For the children who were assessed to have regressed, the reasons for this are unclear, but could conceivably reflect aspects of the child’s development including co-occurring conditions, or could be an artefact of testing (e.g., variability in engagement in tasks at the two timepoints). Further research is needed to explore the variability in children’s trajectories through measurement at multiple timepoints.

The magnitude of change identified in the current cohort of children should be contextualised for several reasons. First, the Spoken Language Benchmarks require children to meet the criteria for a given phase level across all four language domains (phonology, vocabulary, grammar, and pragmatics). However, children on the autism spectrum often demonstrate uneven profiles of language development (Ellawadi & Ellis Weismer, 2015); hence, a lack of change in phase level may mask more subtle increases (or decrease) in skills in a particular domain. Second, the time period between assessments was relatively short, particularly for children who entered the early intervention programs part-way into the year, meaning that the measurement may not have been sensitive to change. Third, the phases of the Spoken Language Benchmarks are sequential, but the nature and level of skill acquisition required to move from one phase to the next varies. To illustrate, grammar is only assessed from Phase 2 onwards. Furthermore, the Spoken Language Benchmarks are based on the phases of acquisition in neurotypical children, which may not be linear for children on the autism spectrum. Fourth, it is expected that a multitude of factors influenced children’s progression (or lack thereof), including the characteristics of any interventions received. In this regard, and while acknowledging the limitation, the close examination of within cohort changes has the potential to shed light on clinically relevant factors that may influence children’s spoken language outcomes.

Our second aim was to examine nine factors that clinicians proposed may account for differences in spoken language acquisition amongst preschool aged children who enter early intervention services with minimal verbal language. The model comprising seven factors (functional use of objects and ratio of speech to non-speech behaviours excluded due to multicollinearity) was significant and explained 64% of the variance in spoken language outcomes (i.e., acquisition of Phase 3 spoken language) with high sensitivity indicating the relevance of the predictors. The direction of effect for each variable was consistent with hypotheses posed. Younger chronological age, higher receptive language scores, and fewer autism characteristics were associated with increases in spoken language. Not surprisingly, but importantly, the findings point to the relevance of clinical insight when it comes to better understanding the heterogenous developmental profiles and outcomes for children on the autism spectrum (citation withheld for blind review). With these considerations in mind, the findings point to the importance of clinicians applying, and sharing with colleagues, their clinical insights when making recommendations for interventions for children on the autism spectrum within an EBP framework. Furthermore, the findings should serve to encourage researchers to collaborate with clinicians in developing and testing hypotheses.

Despite the overall significance of the model, none of the seven predictors was individually significant, which demonstrates the interconnected nature of the skills examined. However, children’s *rate of communicative acts* at Time 1 showed a large effect size and warrants consideration. From a clinical perspective, the finding appears to reinforce the importance of creating learning environments that foster children’s motivation to spontaneously engage in communicative acts as the foundation for spoken language and broader communication development (Paul, 2008). In making this observation, we note that a child’s rate of communicative acts will rarely be independent of a range of other factors, such as the number, quality, and consistency of communication opportunities provided by the communication partner and their responses to the child’s communicative attempts. Nevertheless, and importantly, the variable was derived from an existing clinical assessment tool (CSBS-DP) in which children are offered a consistent set of communicative temptations (i.e., situations designed to be enticing to communicate to access activities or materials). The presentation of communication opportunities in this manner may help to differentiate the relative influence of the child’s own propensity to communicate from the influence of the communication partners’ behaviour. Furthermore, it appears that a clinically feasible, play-based task may not only help to characterise children’s communication as part of assessment of skills and goal setting, but also to predict which children are likely to experience a more favourable outcome. From a research perspective, there is currently concerted effort to identify variables that may help to match and personalise interventions for individual children on the autism spectrum (e.g., Lin et al., 2021; Mouga et al., 2020; Pecukonis et al., 2019). Our findings of the potential relevance of the rate of communicative acts, which are consistent with those reported in earlier studies (e.g., Plumb & Wetherby, 2013; Shumway & Wetherby, 2009), suggest it could be a worthy candidate for inclusion in algorithms designed to support clinical decision-making.

### Limitations and Future Directions

The findings of this study should be considered in the context of several limitations, each of which also has implications for future research. First, this was a prospective cohort study with children attending community-based early intervention centres, not a clinical trial. There was no control group and children were exposed to different interventions. Accordingly, the analysis related to children’s change within programs, without attention to intervention effects. There is a need for more consistent examination and reporting of spoken language outcomes, and factors that may moderate these, in the context of clinical trials where the influence of the interventions can also be assessed.

Second, in selecting the putative predictors and corresponding measurement tools, priority was given to clinically relevant tools and, where possible, those already commonly used in practice. Therefore, for example, the rate of communicative acts was calculated on the basis of the three standardised trials rather than a more comprehensive measure across the assessment session. In research of this kind, there is often a balance to be struck between the use of clinically feasible measures and those with potentially more precision, but that are not readily amenable to clinical application. We would encourage, where possible, greater consistency in the measurement of variables of interest across studies, including clinician-derived data from standardised tools. A broader consideration is that factors were selected for examination in this study via a clinically-focused, as opposed to data-driven, process. Two key considerations are (a) that a different team of clinicians and researchers may have prioritised a different set of factors; and (b) relatedly, the adoption of a data-driven approach (e.g., principal component analysis) would allow for replication of the process by which variables were selected.

Third, the assessments were usually completed in one session, and by research assistants who were not familiar communication partners. These methods, which were selected to help increase rigor, may nevertheless pose challenges in attempts to conduct comprehensive assessments of the communication skills of children with minimal spoken language. Communication between children and adults is co-constructed, and so it is possible that children would have demonstrated different repertoires of skills if interacting with a familiar adult. Furthermore, in practice, clinicians collect information over time, including via dynamic assessment, which focuses less on the child’s existing skills and more on the child’s development of skills when provided with new learning opportunities. Future studies could include examination of children’s skills with both familiar and unfamiliar communication partners, thereby helping to account for the important influence of partners on children’s communication, learning, and participation; as well as examination of changes in children’s skills when provided with new learning opportunities, such as through the provision of AAC.

Fourth, the current study focused on spoken language outcomes. However, we acknowledge that aspects of children’s development do not occur in isolation. For example, communication may be considered within the context of broader cognitive development and any developmental delay in fine motor skills may impact children’s performance in assessment tasks. A further consideration is the critical question of whether the spoken language changes observed, and factors predicting these, may be unique to children on the autism spectrum, or instead are relevant to a broader group of children with neurodevelopmental conditions, such as intellectual disability and developmental language disorder. As stated at the outset, children on the autism spectrum should be supported and encouraged to use a range of communication modes of their choosing, which may include AAC. It is important that future studies examine the potential moderating effects of AAC on children’s spoken language outcomes, as well as changes in children’s broader communication including the use of AAC.

Finally, consideration should be given to two aspects of the sample. First, the lack of detailed demographic characteristic information is a clear limitation of the study. Relevant factors to consider include children’s race, culture, socio-economic status, home language environment (e.g., mono-lingual, bi/multi-lingual), and co-occurring conditions (e.g., epilepsy, intellectual disability). This information would have been helpful in contextualising the study, including describing the participants and interpreting the findings. Second, the relatively small sample size, which, although not uncommon in clinical research (e.g., Pecukonis et al., 2019; Saul & Norbury, 2020; Yoder et al., 2015), limits both power and generalization of the findings. In terms of power, findings could inform a priori calculations for future larger studies into whether predictors (especially the rate of communicative acts, which appears most promising) are indeed unique predictors. Further, larger more diverse samples would allow more fine-grained analysis of potential subgroups/profiles in terms of response to intervention and stepwise changes to better inform intervention efforts moving into the future. Such research may be achieved through greater international collaboration efforts and multi-site pooling of standardised protocols to advance the field.

## Conclusion

The findings from this study contribute to existing evidence of the clinical heterogeneity in preschool-aged children on the autism spectrum, in this case in terms of their spoken language trajectories while attending community-based intervention services. A set of seven clinician-proposed child-related factors—selected on the basis of their theoretical relevance, prior empirical evidence, and identified relevance to clinical practice—were found to account for over half of the variance in spoken language change for the children in this study. Of the seven predictors, none were statistically significant, but children’s rate of communicative acts had a large effect size. The findings point to the complex nature of spoken language development in children on the autism spectrum, highlight the value of clinical insight, and reinforce the need for concerted clinical, research, and community effort to address the unmet needs of a large minority of children.
