# Correction to: Spoken Language Change in Children on the Autism Spectrum Receiving Community-Based Interventions

**DOI:** 10.1007/s10803-022-05633-9

**Published:** 2022-06-18

**Authors:** David Trembath, Matt Stainer, Teena Caithness, Cheryl Dissanayake, Valsamma Eapen, Kathryn Fordyce, Veronica Frewer, Grace Frost, Kristelle Hudry, Teresa Iacono, Nicole Mahler, Anne Masi, Jessica Paynter, Katherine Pye, Shannon Quan, Leanne Shellshear, Rebecca Sutherland, Stephanie Sievers, Abirami Thirumanickam, Marleen F. Westerveld, Madonna Tucker

**Affiliations:** 1 Griffith University, Southport, QLD Australia; 2 La Trobe University, Bundoora, VIC Australia; 3 University of New South Wales, Sydney, NSW Australia; 4 St Giles Society, Launceston, TAS Australia; 5 Murdoch Children’s Research Institute, Melbourne, VIC Australia; 6 Anglicare, Prospect, SA Australia; 7 University of Canberra, Bruce, ACT Australia; 8 University of Adelaide, Adelaide, SA Australia; 9 Griffith University, Griffith Institute for Educational Research, Mount Gravatt, QLD Australia; 10 AEIOU, Woolloongabba, QLD Australia

## Correction to: Journal of Autism and Developmental Disorders 10.1007/s10803-022-05511-4

Please note the following corrections to this article:

Two citations are now provided:

- Under the heading *Functional Use of Objects*: “…pretend/imaginative (citation withheld for blind review).” should say “…pretend/imaginative (Rose et al., 2020).”
- Under the heading *Discussion*: “…children on the autism spectrum (citation withheld for blind review).” should say “…children on the autism spectrum (Trembath et al., 2021).”

Typographical errors that are present but that do not alter interpretation of the findings are acknowledged.

